# Imbalanced Decision Hierarchy in Addicts Emerging from Drug-Hijacked Dopamine Spiraling Circuit

**DOI:** 10.1371/journal.pone.0061489

**Published:** 2013-04-24

**Authors:** Mehdi Keramati, Boris Gutkin

**Affiliations:** Group for Neural Theory, INSERM U960, Departément des Etudes Cognitives, Ecole Normale Supérieure, Paris, France; Tulane University Medical School, United States of America

## Abstract

Despite explicitly wanting to quit, long-term addicts find themselves powerless to resist drugs, despite knowing that drug-taking may be a harmful course of action. Such inconsistency between the explicit knowledge of negative consequences and the compulsive behavioral patterns represents a cognitive/behavioral conflict that is a central characteristic of addiction. Neurobiologically, differential cue-induced activity in distinct striatal subregions, as well as the dopamine connectivity spiraling from ventral striatal regions to the dorsal regions, play critical roles in compulsive drug seeking. However, the functional mechanism that integrates these neuropharmacological observations with the above-mentioned cognitive/behavioral conflict is unknown. Here we provide a formal computational explanation for the drug-induced cognitive inconsistency that is apparent in the addicts' “self-described mistake”. We show that addictive drugs gradually produce a motivational bias toward drug-seeking at low-level habitual decision processes, despite the low abstract cognitive valuation of this behavior. This pathology emerges within the hierarchical reinforcement learning framework when chronic exposure to the drug pharmacologically produces pathologicaly persistent phasic dopamine signals. Thereby the drug hijacks the dopaminergic spirals that cascade the reinforcement signals down the ventro-dorsal cortico-striatal hierarchy. Neurobiologically, our theory accounts for rapid development of drug cue-elicited dopamine efflux in the ventral striatum and a delayed response in the dorsal striatum. Our theory also shows how this response pattern depends critically on the dopamine spiraling circuitry. Behaviorally, our framework explains gradual insensitivity of drug-seeking to drug-associated punishments, the blocking phenomenon for drug outcomes, and the persistent preference for drugs over natural rewards by addicts. The model suggests testable predictions and beyond that, sets the stage for a view of addiction as a pathology of hierarchical decision-making processes. This view is complementary to the traditional interpretation of addiction as interaction between habitual and goal-directed decision systems.

## Introduction

“We admitted we were powerless over our addiction—that our lives had become unmanageable” states the very first tenet of the Narcotics Anonymous 12-step program [1]. This spotlights how powerless addicts find themselves when it comes to resisting drugs, despite knowing that drug-taking is a wrong course of action [2]–[4]. In fact, the hallmark of addiction is compulsive seeking of the drugs even at the cost of evident adverse consequences [5]. A signature of such pathological behavior becomes evident in controlled experiments where addicts exhibit a characteristic “self-described mistake”: an inconsistency between the potent behavioral response toward drug-associated choices and the relatively low subjective value that the addict reports for the drug [4], [6], [7]. When combined with the loss of inhibitory cognitive control over behavior, after protracted exposure to drugs, this divergence between the cognitive plans and the consolidated habits may result in a transition from casual to compulsive drug-seeking behavior [8].

The loss of cognitive control and self-described mistake have so far eluded a principled explanation by formal models of addiction [9]–[13]. Previous computational theories of drug addiction, mostly posed within the reinforcement learning framework, view addiction as a pathological state of the habit learning (stimulus-response) system [9]–[13]. The central hypothesis behind all those models is that the pharmacological effect of drugs on dopamine signaling, supposedly carrying a stimulus-response teaching signal, results in gradual over-reinforcement of such associations. This effect in turn leads to compulsive drug-seeking habits. While this reduced view of addiction has captured some aspects of the phenomenon, a growing consensus in the addiction literature indicates that multiple learning systems are involved in the pathology. Only such a more complex picture that includes brain's cognitive, as well as low-level habitual processes, can explain the variety of addiction-like behaviors [8], [14].

In this paper, we adopt a hierarchical reinforcement learning approach [15] where decisions are represented at different levels of abstraction, in a cognitive-to-motor hierarchy. We assume that a cascade of dopamine-dependent learning signals links levels of the hierarchy together [16]. We further assume that drugs of abuse pharmacologically hijack the communication mechanism between levels of abstraction. Based on these assumptions, we show that the reported cognitive dissonance in addicts emerges within the hierarchical reinforcement learning framework when chronic drug-exposure disrupts value-learning across the decision hierarchy. This disruption results in a pathological over-valuation of drug choices at low-level habitual processes and hence drives habitual drug-seeking behavior. We then demonstrate that “disliked” but compulsive drug-seeking can be explained as drug-hijacked low-level habitual processes dominating behavior, while healthy cognitive systems at the top representational levels lose control over behavior. Furthermore, we demonstrate that the proposed model can account for recent evidence on rapid vs. delayed development of drug cue-elicited dopamine efflux in the ventral vs. dorsal striatum, respectively, as well as the dependence of this pattern on dopamine spiraling circuitry.

## Materials and Methods

### Preliminaries

**Figure 1:**
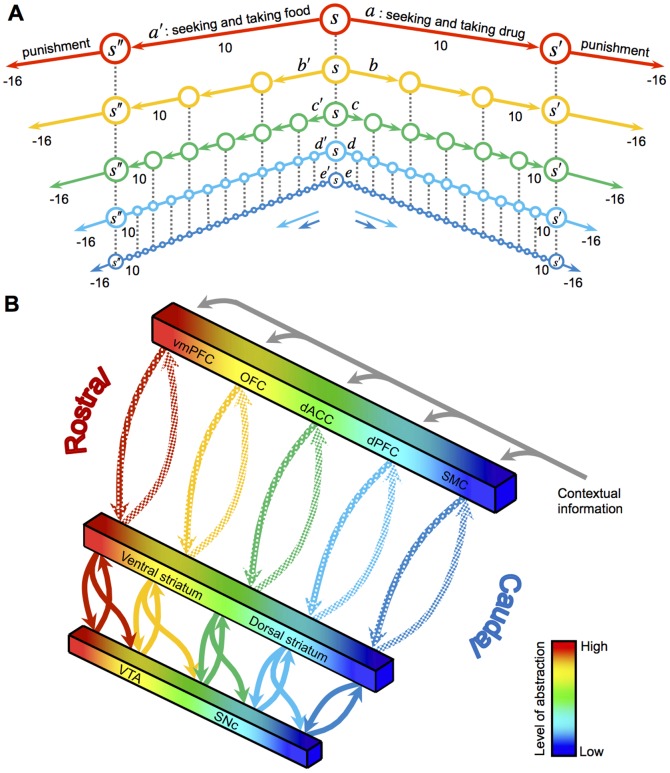
Hierarchical organization of behavior and the cortico-BG circuit. **A**, An example of a decision hierarchy for two alternative choices: drug vs. food. Each course of action is represented at different levels of abstraction, supposedly encoded at different cortico-BG loops. Seeking each of the two types of reward might follow a punishment of magnitude 16. **B**, Glutamatergic connections from different prefrontal areas project to striatal subregions and then project back to the PFC through the pallidum and thalamus, forming several parallel loops. Through the striato-nigro-striatal dopamine network, the ventral regions of the striatum influence the more dorsal regions. vmPFC, ventral medial prefrontal cortex; OFC, orbital frontal cortex; dACC, dorsal anterior cingulate cortex; SMC, sensory-motor cortex; VTA, ventral tegmental area; SNc, substantia nigra pars compacta. **Figure 1B** Modified from ref 21.

In concordance with a rich cognitive psychology literature, our hierarchical reinforcement learning [15], [18] framework assumes that an abstract cognitive plan like “brewing tea” can be broken into a sequence of lower-level actions: boiling water, putting tea in the pot, etc. Such decomposition proceeds until concrete motor-level responses at the lowest level of the hierarchy (Figure 1A). Neurobiologically, the different levels of decision hierarchy from cognitive to motor levels are represented along the rostro-caudal axis of the cortico-basal ganglia (BG) circuit [19]–[21]. This circuit is composed of several parallel closed loops between the frontal cortex and the basal ganglia [22], [23] (Figure 1B). Whereas the anterior loops underlie more abstract representation of actions, the caudal loops, consisting of sensory-motor cortex and dorsolateral striatum, encode low-level habits [19]–[21].

Within this circuitry, the phasic activity of midbrain dopamine (DA) neurons projecting to the striatum signals the error between predicted and received rewards, thereby carrying stimulus-response reinforcing information [24]. These DAergic projections form a cascading serial connectivity linking the more ventral regions of the striatum to progressively more dorsal regions through the so-called ″spiraling″ connections [25]–[27] (Figure 1B). Functionally, such feed-forward organization connecting the rostral to caudal cortico-BG loops allows directed coupling from coarse to fine representations. Accordingly, the DA spirals are hypothesized to provide a neurobiological substrate for the progressive tuning of the reward prediction error by the higher levels of the hierarchy (encoding the abstract knowledge about the value of behavioral options). This error is then utilized for updating action-values at more detailed levels [16]. In other words, the DA spirals allow for the abstract cognitive levels of valuation to guide the learning in the more detailed action-valuation processes.

### Theory sketch

In terms of the computational theory of reinforcement learning [28] (RL), the agent (in our case a person or an animal) learns to make informed action-choices by updating its prior estimated value, 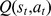, for each state-action pair, 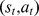, when a reward 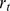 is received by the agent at time 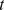 as a result of performing an action 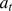 in the contextual state (stimulus) 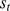. The value 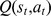 is updated by computing the reward prediction error signal. This signal not only depends on the instantaneously received reward (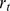), but also on the value of the new state the agent ends up in, after that action has been performed. Denoted by 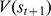, this temporally-advanced value-function represents the sum of future rewards the animal expects to receive from the resultant state, 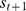, onward. The prediction error can be computed by the following equation:



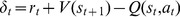
(1)


Intuitively, the prediction error signal computes the discrepancy between the expected and the realized rewarding value of an action. In a hierarchical decision structure, however, rather than learning the 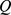-values independently at different levels, more abstract levels can tune the teaching signal computed at lower levels. Since higher levels of the hierarchy represent a more abstract representation of environmental contingencies, learning occurs faster in those levels. This is due to the relative low-dimensionality of the abstract representation of behavior: an action plan can be represented as a single step (one dimension) at the top level of the hierarchy and as multiple detailed actions (multiple dimensions) at the lower levels of the hierarchy. The top level value of this action-plan would be learned quickly as compared to the detailed levels where the reward errors would need to back-propagate all the detailed action-steps. Thus, tuning the lower level values by the value information from the higher levels can speed up the convergence of these values. One statistically efficient way of doing so is to suppose that for computing the prediction error signal at the 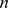-th level of abstraction, 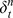, the temporally-advanced value function, 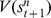, comes from one higher level of abstraction, 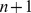 [16]:



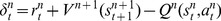
(2)


To preserve optimality, equation 2 can be used for computing the prediction error only when the last constituent primitive action of an abstract option is performed (see Figure S1 in File S1). In other cases, value-learning at different levels occur independently, as in equation 1. In both cases, the teaching signal is then used for updating the prior values at the corresponding level:



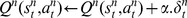
(3)where 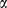 is the learning rate. This form of inter-level information-sharing is biologically plausible since it reflects the spiraling structure of the DA circuitry, carrying the information down the hierarchy in the ventro-dorsal direction. At the same time, being guided by more abstract levels significantly accelerates learning, alleviating the high-dimensionality of value learning at detailed levels [16].

In this paper we show that the interaction between a modified version of the model developed in [16] and the specific pharmacological effects of drugs of abuse on the dopaminergic system can capture addiction-related data at radically different scales of analysis: behavioral and circuit-level neurobiological. First, the new model brings about a possible cogent explanation for several intriguing behavioral aspects associated with addiction to drugs (e.g. the self-described mistake [4], [6], [7]). Second, we can account for a wide range of evidence regarding the dynamics of the drug-evoked dopamine release [17].

We modify the model presented in [16] as follows. We make the model more efficient in terms of working memory capacity by replacing 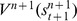 with 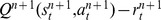, in equation 2, since the two values converge to the same steady level (see Figure S2 in File S1, for computational and neurobiological basis):



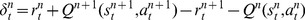
(4)


Here, 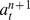 is the relatively abstract option and 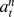 is the last primitive action in the behavioral sequence that full-fills this option. Similarly,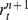 is the rewarding value of 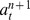, which includes 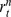 (the rewarding value of 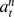).

Crucially, the various drugs abused by humans share a fundamental property of pharmacologically increasing dopamine concentration within the striatum [29]. Accordingly, we incorporate this pharmacological effect of the drug by adding a positive bias, 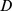, (see also [9]–[12]) to the prediction error signal carried by dopamine neurons (see Figure S3 in File S1, for computational and neurobiological basis):



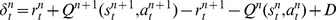
(5)


Here 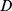 captures the direct pharmacological effect of drug on the DA system, and 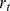 is its reinforcing value due to the euphorigenic effects (see File S1 for supplementary information).

While equations 3 and 5 together define the computational mechanism to update the values in our model, we also hypothesize that an uncertainty-based competition mechanism determines the level of abstraction that controls behavior. This is inspired by the mechanism proposed in [29] for arbitration between the habitual and goal-directed systems. In this respect, at each decision point, only the level of abstraction with the highest certainty in estimating the value of choices controls behavior. Once this level has made the decision to act, all the lower levels of the hierarchy will be deployed by this dominant level to implement the selected action as a sequence of primitive motor responses (see File S1 for supplementary information; Figure S4 in File S1; Figure S5 in File S1). Upon receiving the reward feedback from the environment, the values at all the levels are updated. This uncertainty-based arbitration mechanism predicts that as abstract processes are more flexible, they have superior value-approximation capability during the early stages of learning and thus, control behavior at these stages. However, since the abstract levels use a coarse representation of the environment (e.g. due to containing a relatively small number of basis functions), their ultimate value approximation capability is not as precise as those of detailed levels. In other words, after extensive training the certainty associated with the estimated values is lower for the lower levels of the hierarchy as compared to the upper levels. Thus, with progressive learning, the lower levels of the hierarchy take over the control over the action selection, as their uncertainty decreases gradually. This is in agreement with several lines of evidence showing a progressive dominance of the dorsal over the ventral striatum in the control over drug-seeking (as well as seeking natural rewards) [8], [30], [31].

## Results

### Hierarchy valuation inconsistency emerges under drug but not natural rewards

**Figure 2:**
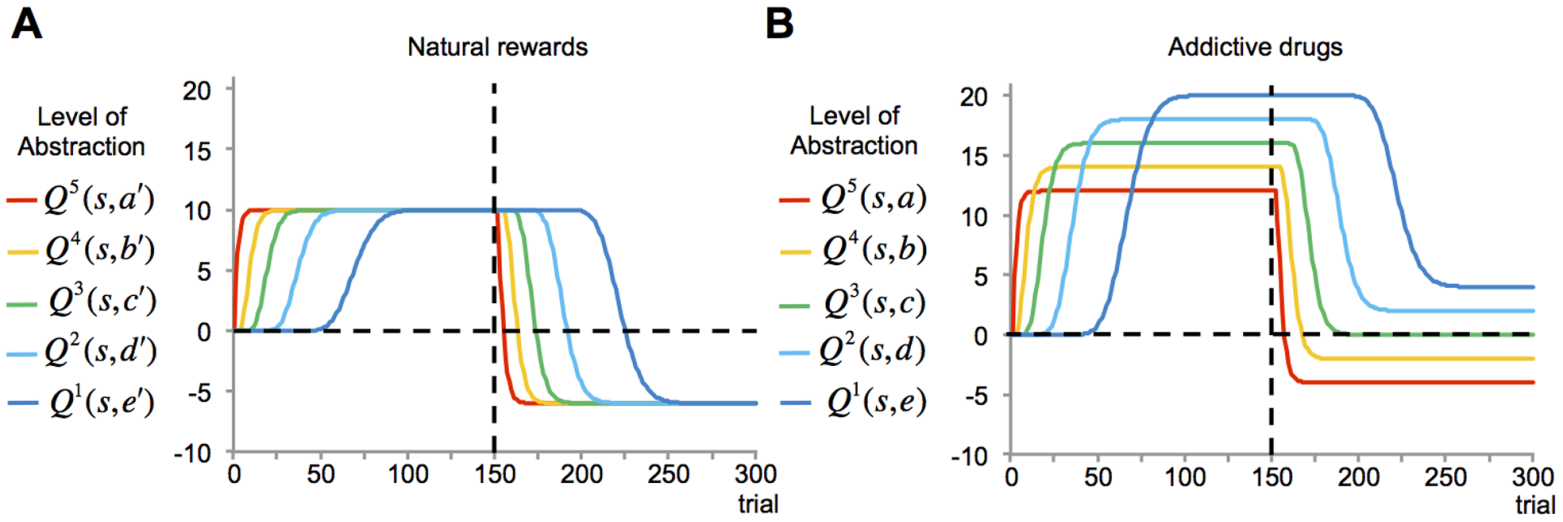
Motivation for food vs. drug at different levels of abstraction (simulation results). In the first 150 trials where no punishment follows the reward, the value of seeking natural rewards at all levels converge to 10 (**A**). For the case of drug, however, the direct pharmacological effect of drug (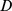, set to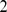) results in the asymptotic value at each level to be 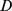 units higher than that of one higher level of abstraction (**B**). Thus, when followed by punishment, whereas cognitive loops correctly assign a negative value to drug-seeking choice, motor-level loops find drug-seeking desirable (positive value). The curves in this figure show the evolution of values in “one” simulated animal and thus, no statistical analysis was applicable.

In contrast to the previous reinforcement learning-based computational models of addiction [9]–[13] which are based on a single-decision-system approach, our account is build upon a multiple-interacting-systems framework. As a result, although the form of modeling drug's effect on the prediction error signal in our model is similar to the previous ones [9]–[12], it results in fundamentally different consequences. The drug-induced transient dopamine increase boosts the immediate prediction error at each level of the hierarchy and as a result, entrains a bias, 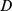, on the transfer of knowledge from one level of abstraction to the next, along the coarse-to-fine direction of the hierarchy. This bias causes the asymptotic value of drug-seeking at a given level to be 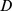 units higher than that of one more abstract layer (Figure 2B). The accumulation of these discrepancies along the rostro-caudal axis progressively induces significant differences in the value of drug-seeking behaviors between the top and bottom extremes of the hierarchy. Thus, even when followed by a strong punishment, the value of drug-associated behavior remains positive at the low-level motor loops, while it becomes negative at cognitive levels. In other words, the model predicts that accumulation of drug effect over DA spirals increases drug-seeking value at motor-level habits to such high amplitude that even a strong natural punishment will not be able to decrease it sufficiently. We suggest that this explains the inconsistency between cognitive and low-level evaluation of drug-related behaviors in addicts. In other words, we propose that compulsive drug seeking and the significantly reduced elasticity to associated costs stems from the pharmacological effect of the drug hijacking the dopamine-dependent mechanism that transfers the information among the levels of decision hierarchy.

While drugs, in our model, result in imbalanced valuation across levels, the value of natural rewards converges to the same value across all levels, due to lack of a direct pharmacological effect on DA signaling mechanism (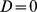). Consequently, neither inconsistency nor overvaluation at detailed levels will be observed for the case of natural rewards (Figure 2A). Overvaluation of drug-seeking responses at lower levels of the hierarchy should result in abnormal preference of drugs over natural rewards, and over-engagement in drug-related activities.

### Differential dopamine responding in the ventral versus dorsal striatum to drug-associated cues

Neurobiologically, differential roles of the striatal subregions in the acquisition and expression of drug-seeking behavior has taken center stage in addiction research. Converging evidence from different lines of research suggests that the behavioral transition from recreational to compulsive drug use reflects a neurobiological shift of valuation from the ventral to the dorsolateral striatum [8], [33], [34], corresponding to a shift from cognitive to detailed levels in our model. Consistent with our model, DA spiraling network connecting the ventral to progressively more dorsal regions of the striatum is shown to play a pivotal role in this transition [25].

In a key recent study Willuhn et al. [17] assessed the pattern of dopamine release in response to drug-associated cues in the ventral and dorsolateral striatum of rats during three weeks of experiencing cocaine. Using fast-scan cyclic voltammetry, the critical observation was that cue-induced DA efflux in the ventral striatum emerges even after very limited training. In contrast, the dorsolateral striatum showed cue-triggered DA efflux only after extensive training, and the development of this release pattern disappeared when the ventral striatum was lesioned in the ipsilateral hemisphere.

Since the temporal resolution of fast-scan voltammetry captures subsecond fluctuations in concentration, the observed pattern of DA efflux should be attributed to “phasic” DA signaling and thus, to the prediction error signal, according to the RL theory of dopamine [24]. According to RL theory, the prediction error signal upon observing an unexpected stimulus is equal to the rewarding value that that stimulus predicts. Therefore, cue-induced DA release is equivalent to the value predicted by that cue.

**Figure 3:**
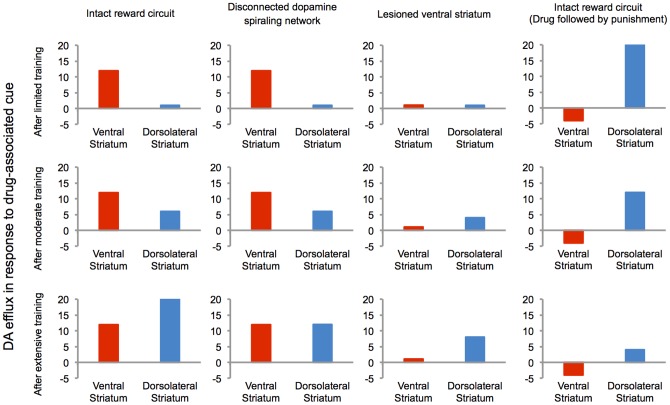
Dopamine efflux at different striatal subregions in response to drug-associated cues (simulation results). In line with experimental data [17], the model shows (left column) that in response to drug-associated cues, there will be dopamine efflux in the ventral striatum, after limited and extensive training. In more dorsolateral subregions, however, cue-elicited DA efflux will develop gradually during the course of learning. The model predicts (second column from right) that this delayed development of cue-elicited DA efflux in dorsal striatum depends on the DA-dependent serial connectivity that links the ventral to the dorsal striatum. That is, as a result of disconnecting the DA spirals, whereas cue-elicited DA response remains intact in the ventral striatum, it significantly decreases in the dorsolateral striatum. Moreover, the model predicts (third column from right) similar results for cue-induced DA efflux in dorsolateral striatum for the case of lesioned ventral striatum. Finally, if after extensive drug-cue pairing in intact animals, a punishment follows drug, the model predicts (right column) that drug-related cue results in inhibition of the ventral leg of DA spirals, even after limited training. In more dorsal regions, however, DA efflux decreases slowly during learning, but will remain positive, even after extensive drug-punishment pairing. The data presented in this figure are obtained from “one” simulated animal and thus, no statistical analysis was applicable.

In this respect, our hierarchical framework provides a formal explanation for the differential pattern of ventral versus dorsal striatal DA efflux reported in [17]. The value predicted by the drug-associated cue at the abstract cognitive levels of the hierarchy increases rapidly at the very early stages of training (Figure 2B), due to low-dimensionality of the learning problem at high levels of abstraction. As a result, our model shows that the cue-induced DA efflux should be observed in the ventral striatum even after limited training (Figure 3). At the more detailed levels of representation, however, the learning process is slow (Figure 2B), due to high-dimensionality of the problem space, as well as dependency of learning on more abstract levels through DA spirals. Consequently, cue-induced DA efflux in the dorsolateral striatum should develop gradually and become observable only after extensive training (Figure 3).

Furthermore, our model explains the evidence in [17] that such delayed development of cue-elicited DA efflux in the dorsolateral striatum depends on the ventral striatum (Figure 3). In our model, a simulated unilateral lesion of the ventral striatum (the abstract valuation level in the model) significantly decreases the drug cue-predicted value at detailed levels in the ipsilateral hemisphere and thus, significantly decreases the level of cue-induced DA efflux. In order to model lesion of the ventral striatum, we simply fix the value of all stimuli at the highest level of the hierarchy to zero.

Similarly, our model predicts that the development of phasic DA signaling in the dorsolateral striatum depends on the integrity of the DA spiraling circuit (Figure 3). In fact, a disconnection in the DA spiraling circuit in our model cuts the communication across levels of abstraction, which in turn, prevents accumulation of the drug-induced bias on the reinforcement signal, along the levels of decision hierarchy. To model the disconnection in the DA-dependent serial circuitry of ventral to dorsal striatum, we clamp each level of abstraction to compute the prediction error signal locally (as in equation 3), without receiving the value of the temporally advanced state from the immediately higher level of abstraction.

Furthermore, the model predicts that the pattern of cue-elicited DA efflux will change if after an extensive training with cocaine and cocaine associated cues, as in the above experiment, one starts to pair the cocaine delivery with a strong punishment. We predict that the DA efflux in response to the cocaine-associated cue should rapidly decrease below baseline in the ventral striatum. In the dorsolateral striatum, however, cue-induced DA release should stay above baseline (Figure 3) with a possible delayed partial decrease. This indicates assigning positive subjective value to the drug stimulus at detailed levels, despite negative (below baseline) values at cognitive levels. It is noteworthy that this prediction depends on the assumption that punishment is treated by the brain simply as a negative reward. This assumption is somewhat controversial: it is clearly supported by experimental studies [35], yet also discussed otherwise by others [14], [36]. Except for this prediction, other aspects of the model do not depend on whether punishment is encoded by dopamine or by another signaling system.

The training regimen used by Willuhn et al. [34] is not sufficiently extended to producing compulsive drug-seeking behavior, characterized by insensitivity to drug-associated punishments [37], [38]. Thus, a key question to be answered is what is the relation between delayed development of cue-induced DA response in DLS, and late development of compulsive responding. According to our model, compulsive behavior requires not only the excessive valuation of drug choice at low levels of the hierarchy, but also the transfer of control over behavior from the abstract cognitive to the low-level habitual processes. The time scale of these two processes are only partly dependent to each other: the over-valuation process depends on the prediction error signal, while the transfer of behavioral control also depends on the relative uncertainties in value-estimation. Hence, the over- valuation of drug-associated cues at low levels of the hierarchy can precede the shift of control over behavior from top to the bottom of the hierarchy. The exact time scales of the two processes depend on the learning rate and the noise inherent at the different levels, respectively (see File S1 for supplementary information). In other words, it is likely that the cue-induced dopamine efflux in the DLS may develop significantly before the compulsive drug-seeking is behaviorally manifested.

### Behavioral implications of the inconsistent valuation for drugs versus natural rewards

Behaviorally, in our model, if punishment is paired with drug at the early stages of voluntary drug use, the abstract value of drug-seeking response becomes negative rapidly. Assuming that drug-seeking is controlled by abstract levels during these early stages, negative abstract evaluation of drug choice makes the subject unwilling to experience that course of action any longer. This will prevent consolidation of strong low-level preference toward drugs over time. Thus, the model explains elasticity of drug choices to costs during the early stages of drug consumption, but not after chronic use. Consistently, animal models of addiction show that insensitivity of drug-seeking responses to harmful consequences associated with drug develops only after prolonged drug self-administration, but not limited drug use [37], [38]. In contrast to our theory, earlier computational models of addiction [9], [10] are in direct contradiction with this body of evidence, since they predict that adverse behavioral outcomes that immediately follow drug use, have no motivational effect even at the very early stages of experiencing drugs (see File S1 for supplementary information).

**Figure 4:**
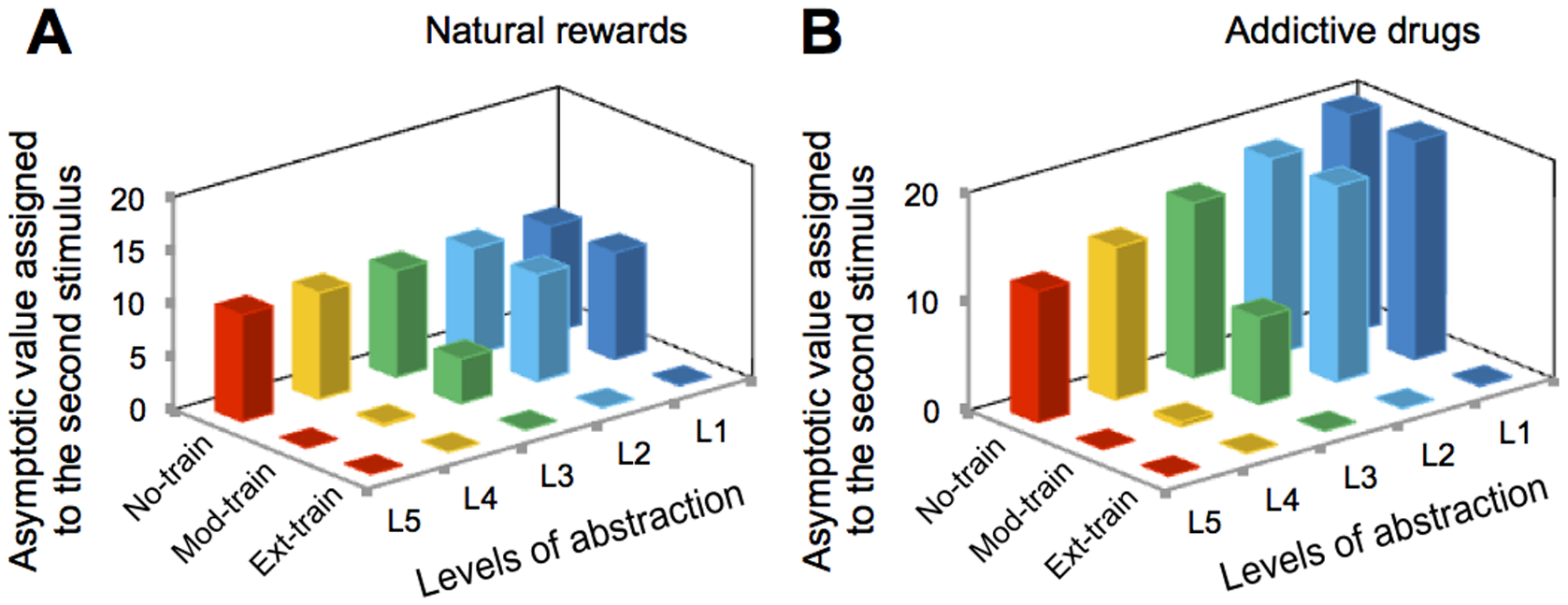
Blocking effect for natural vs. drug rewards. The model predicts that blocking occurs for natural rewards (**A**) and drugs (**B**), only if the initial training period is “extensive”, such that the first stimulus fully predicts the value of the outcome. After “moderate” training, cognitive levels that are more flexible fully predict the values and thus, block further learning. However, learning is still active in low-level processes when the second training phase (simultaneous presentation of both stimuli) starts. Thus, our model predicts that moderate initial training in a blocking experiment with natural rewards will also result in cognitive/behavioral inconsistency. The data presented in this figure are obtained from “one” simulated animal and thus, no statistical analysis was applicable.

Our model further accounts for the occurrence of blocking effect for drug outcomes [39]. Blocking is a conditioning phenomenon where prior pairing of a stimulus A with an outcome blocks formation of association between a different stimulus B with that outcome in a subsequent training phase, where both A and B are presented before the delivery of the outcome [40]. Results of simulating our model in a Pavlovian experimental design (see File S1 for supplementary information on the Pavlovian version of the model) shows that for both cases of natural rewards and drugs, when the estimated value at a certain level of the hierarchy reaches its steady state (rather than growing unboundedly), no further learning occurs at that level, since the prediction error signal has decreased to zero (Figure 4). Thus, associating a new stimulus with the already-predicted reward will be blocked. Behavioral evidence showing a blocking effect associated with both drug and natural reinforcers [39] has been used as a major argument to criticize the previously proposed dopamine-based computational model of addiction [9]. Here we showed that focusing on the hierarchical nature of representations and dorsal-ventral spiraling dopamine loop organization can in fact account for the blocking data, thereby circumventing this criticism (see File S1 for supplementary information).

As mentioned before, several lines of evidence show a progressive dominance of the dorsal over the ventral striatum in the control over behavior during the course of learning [8], [31], [32]. Being interpreted on a background of those evidence, the imbalanced drug-seeking valuation across the hierarchy also explains addicts' unsuccessful efforts to cut down drug-use after prolonged experience with drug, when control over drug-related choices has shifted from cognitive to low-level habitual processes. This supremacy of drug-dominated processes naturally leads to behavioral inelasticity to drug-associated costs (compulsive drug-seeking), likely accompanied with self-described mistake. For the case of natural rewards, however, our model predicts that even though behavioral inelasticity increases over the course of learning, as no valuation-inconsistency develops across the levels of the hierarchy, punishments associated with reward will eventually inhibit reward-seeking.

Our model focuses on evaluation of actions in a “presumably given” decision hierarchy, and leaves aside how the abstract options and their corresponding low-level subroutines are initially discovered during development. Discovering the decision hierarchy is proposed to be a bottom-up process, accomplished by chunking together sequences of low-level actions and constructing more abstract options [41]. This process, supposedly undergoing a shift from the dorsal to the ventral striatum, is in the opposite direction of the competition mechanism proposed here, for taking control over behavior.

## Discussion

The growing body of evidence on the differential role of different striatal subregions in addiction is usually interpreted in the framework of habitual vs. goal-directed dichotomy [8], [14], [34]. The hierarchical decision making approach we use here is complementary to such dual-system accounts. Whereas the dual-process approach deals with different algorithms (model-free vs. model-base [30]) for solving a single problem, the hierarchical RL framework focuses on different representations of the same problem at different levels of temporal abstraction. In theory, either a habitual or a goal-directed algorithm can solve each of these different representations of the problem. In our model, the accumulation of drug-induced biases over DA spirals occurs in a setting where the value-estimation algorithm is model-free (habit learning). However, this does not rule out existence of model-based systems working at the top levels of the hierarchy. One can simply incorporate the PFC-dependent goal-directed valuation and decision system into the model by assuming that actions at the highest levels of abstraction are evaluated by a goal-directed system. While such complication does not change the nature of results presented in this manuscript, its ensuing additional flexibility in explaining other aspect of addiction is left to future studies. In fact, in our model, irrespective of whether a goal-direct system exists or not, the discrepancy in the asymptotic value of drug-seeking between the two extremes of the hierarchy grows with the number of decision levels governed by the “habitual” process.

In the light of our theory, relapse can be viewed as revival of dormant motor-level maladaptive habits, after a period of dominance of cognitive levels. In fact, one can imagine that as a result of cognitive therapy (in human addicts) or forced extinction (in animal models of abstinence), high value of drug-seeking at the detailed level of the hierarchy is not extinguished, but become dormant due to shift of control back to cognitive levels. Since drug-related behavior is sensitive to adverse consequences at abstract levels, hence drug-seeking can be avoided as long as high-level cognitive processes dominate control of behavior. One can even speculate that the popular 12 step programs (e.g. Alcoholics Anonymous, Narcotics Anonymous, etc) work in part by explicitly requiring the participants to admit the inconsistency of their drug related lifestyle, thereby empowering the abstract cognitive levels to exert explicit control over their behavior. Stressful conditions or re-exposure to drug (priming) can be thought of as risk factors that weaken the dominance of abstract levels over behavior, which can result in re-emergence of drug-seeking responses (due to the latent high non-cognitive values).

In summary, we propose a coherent account for several, apparently disparate phenomena characteristic of drug addiction. Our model provides a normative account for data on the differential roles of the ventral vs. dorsal striatal circuits in drug-seeking acquisition and habit performance, as well as the selective role of feed-forward DA connectivity for effects of drug versus natural reinforcers. Most importantly, we show how the drug-induced pathology in ventral-to-dorsal DA signals trickling the motivational information down cognitive representation hierarchy could leads to discordance between addicts' abstract attitudes toward drug-seeking and what they actually do. Obviously, our model does not, and is not meant to, give a complete account of drug addiction. Explaining other unexplained aspects of addiction requires incorporating many other brain systems that are demonstrated to be affected by drugs of abuse [42]. How to incorporate such systems within the formal computational network remains a topic for further investigation.

## Supporting Information

File S1 **Figure S1,**A sample decision hierarchy with five levels of abstraction. **Figure S2,** The corresponding neural circuit for the three discussed value learning algorithms is a hierarchical decision structure. **A,** Using a simple TD-learning algorithm (equation S7), the prediction error signal in each level of abstraction is computed independently from other levels. **B,** In the model proposed by Haruno and Kawato (4) (equation S8), the value of the temporally-advanced state comes from one higher level of abstraction. **C,** in our model (equation S9) the value of the temporally-advanced state is substituted with a combination of the reward and Q-value of the performed action at a higher level of abstraction. **Figure S3,** Our model predicts different sites of action of drugs on the reward-learning circuit: sites 1 to 3. Drugs affecting sites 4 to 6, in contrast, will not result in the behavioral and neurobiological patterns produced by simulation of the model for drugs, but will produce results similar to the case of natural rewards. **Figure S4,** The task used for simulating the uncertainty-based competition mechanism among the levels of the hierarchy for taking control over behavior. **Figure S5,** Simulation result, showing gradual shift of control over behavior from higher to lower levels of the hierarchy. *Q(s,a)* and *U(s,a)* show the estimated value and uncertainty of the state-action pairs, respectively.
(PDF)
