# Supplementary material for: Imbalanced Decision Hierarchy in Addicts Emerging from Drug-Hijacked Dopamine Spiraling Circuit
Source: PLoS One. 2013 Apr 24;8(4):e61489. doi: 10.1371/journal.pone.0061489 (PMC3634778; doi:10.1371/journal.pone.0061489)
Supplement: File S1 — Figure S1,A sample decision hierarchy with five levels of abstraction. Figure S2, The corresponding neural circuit for the three discussed value learning algorithms is a hierarchical decision structure. A, Using a simple TD-learning algorithm (equation S7), the prediction error signal in each level of abstraction is computed independently from other levels. B, In the model proposed by Haruno and Kawato (4) (equation S8), the value of the temporally-advanced state comes from one higher level of abstraction. C, in our model (equation S9) the value of the temporally-advanced state is substituted with a combination of the reward and Q-value of the performed action at a higher level of abstraction. Figure S3, Our model predicts different sites of action of drugs on the reward-learning circuit: sites 1 to 3. Drugs affecting sites 4 to 6, in contrast, will not result in the behavioral and neurobiological patterns produced by simulation of the model for drugs, but will produce results similar to the case of natural rewards. Figure S4, The task used for simulating the uncertainty-based competition mechanism among the levels of the hierarchy for taking control over behavior. Figure S5, Simulation result, showing gradual shift of control over behavior from higher to lower levels of the hierarchy. Q(s,a) and U(s,a) show the estimated value and uncertainty of the state-action pairs, respectively. (PDF) [file pone.0061489.s001.pdf]

Supplemental Information for

Imbalanced decision hierarchy in addicts emerging from drug-hijacked dopamine spiraling circuit

Mehdi Keramati, Boris Gutkin

Correspondence to: Mr.Mehdi.Keramati@gmail.com

The basic model

Here, we give a more formal description of the hierarchical RL model used in this paper.

We first focus on a "core" Markov decision process that governs the lowest level within the representation hierarchy. Suppose this "core" Markov decision process (MDP) is defined by the 4-tuple $\left\langle S^1, A^1, p_T^1, p_R^1 \right\rangle$, where S^1 is a set of states, A^1 is a set of actions, $p_T^1(s^1 \xrightarrow{a^1} x^1)$ is the transition function, determining the probability of transiting from state s^1 to state x^1 after taking action a^1, and $p_R^1(r^1 | s^1, a^1)$ is the reward function, which determines the probability of receiving reward r^1 from the environment after taking action a^1 in state s^1. The upper indices are not exponents, but refer to the level of abstraction of the corresponding MDP (See Figure S1).

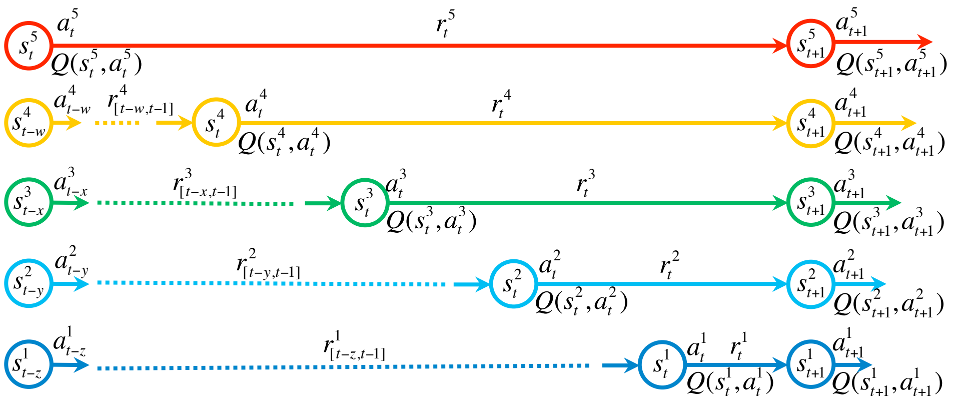

Figure S1: A sample decision hierarchy with five levels of abstraction.

Since we are at the lowest level of the decision hierarchy, each action in this core MDP is called a "primitive action". $\pi^1 = S^1 \times A^1 \rightarrow [0,1]$ is a stationary and stochastic policy, specifying that at time t, the agent performs action a_t^1 with probability $\pi^1(s_t^1, a_t^1)$, whenever it observes state s_t^1. The objective of the agent is to find a policy that will maximize the sum of discounted future rewards from each state. To achieve this objective, the Q-learning algorithm (1) assumes that the agent selects actions based on the subjective values it has assigned to available choices through its past experiences in the environment. These values, denoted by $Q^1(s_t^1, a_t^1)$ for the state-action pair (s_t^1, a_t^1), are aimed to be proportional to the sum of discounted rewards that are expected to be received after taking action a_t^1 onward:

$$Q^1(s_t^1,a_t^1) = E\left[r_t^1 + r_{t+1}^1 + r_{t+2}^1 \ldots \middle| s_t^1, a_t^1\right] = E\left[\sum_{i=t}^{\infty} r_i^1 \middle| s_t^1, a_t^1\right] \quad \text{(S1}$$

Note that for simplicity, we have assumed through this whole manuscript that the discount factor is equal to one. However, the essence of the simulation results does not change for other values of the discount factor. Drug-induced modulation of this variable, supposedly encoded by

serotonin (2), and its contribution to addiction-like behavior is not studied in this work, and is left for future research.

To update the Q-values based on new experiences, the agent computes the discrepancy between the predicted value and the realized reward, each time it receives a reward r_t^1 after taking action a_t^1 in state s_t^1:

$$\delta_t^1 = r_t^1 + V^1(s_{t+1}^1) - Q^1(s_t^1, a_t^1) \quad \text{(S2}$$

$V^1(s_{t+1}^1)$ is the value of the best possible action in the next state: $V^1(s_{t+1}^1) = \max_{a' \in A^1} Q^1(s_{t+1}^1, a')$. The prediction error signal is then used to update the previous estimation:

$$Q^1(s_t^1, a_t^1) \leftarrow Q^1(s_t^1, a_t^1) + \alpha.\delta_t^1 \quad \text{(S3}$$

where α is the learning rate.

We now define a relatively "abstract" MDP (3) above the core MDP, by the 4-tuple $\left\langle S^2, A^2, p_T^2, p_R^2 \right\rangle$, where $S^2 \subseteq S^1$ (See Figure S1). Each abstract action (option), $a^2 \in A^2$, in this abstract MDP is a sequence of primitive actions from the core MDP. For example, in Figure S1, the option a_t^2 is a sequence of some actions from the first level of abstraction, including the primitive action a_t^1.

Formally, an option a^2 can be defined by a triplet $\left\langle I^{a^2}, \pi^{a^2}, \beta^{a^2} \right\rangle$, where I^{a^2} is the input set, determining the states in which option a^2 can be adopted ($I^{a^2} \subseteq S^2 \subseteq S^1$). $\beta^{a^2} : S^1 \rightarrow [0,1]$ is the termination condition, determining the probability that an option will terminate in each state. If the option a^2 is selected to be performed, then primitive actions are selected according to the core level's policy, π^1, until the option terminates stochastically according to β^{a^2}. The policy

π^{a^2} actually follows the same strategy as the policy π^1, meaning that when the abstract system chooses an option, the detailed (core) level implements that option based on its own policy.

Selection among different available options at the abstract level is based on π^2, which is the policy of the abstract system. In order for this system to choose options such that the sum of discounted rewards is maximized, the reward and transition functions of the abstract MDP should be defined as follows. The reward function $p_R^2(r^2 \middle| s^2, a^2)$ should come from the below definition:

$$r^2(s^2,a^2) = E\left[r_t^1 + r_{t+1}^1 + r_{t+2}^1 .. + r_{t+\tau}^1 \middle| \varepsilon(s^2,a^2,\tau)\right] \quad \text{(S4}$$

Where $\varepsilon(s^2,a^2,\tau)$ is the event of a^2 being initiated at time t from state s^2. Furthermore, τ is the random number of time-steps that option a^2 takes to terminate. In Figure S1, for example, r_t^2, which is the reward of action a_t^2, is equal to the sum of rewards acquired during performing the sequence of primitive actions in the core level that constitute the abstract action a_t^2.

Furthermore, the transition function of the abstract MDP should be defined as:

$$p_T^2(s^2 \xrightarrow{a^2} x^2) = \sum_{\tau=1}^{\infty} p(x^2,\tau) \quad \text{(S5}$$

Where $p(x^2,\tau)$ is the is the probability that a^2 terminates in x^2 after τ steps when initiated in s^2. Having defined the reward and transition functions for the abstract MDP, this MDP can be seen as an independent decision problem on which, the Q-learning algorithm can be used to estimate the value of state-option pairs, denoted by $Q^2(s_t^2,a_t^2)$:

$$\delta_t^2 = r_t^2 + V^2(s_{t+1}^2) - Q^2(s_t^2,a_t^2) \quad \text{(S6}$$

$$Q^2(s_t^2,a_t^2) \leftarrow Q^2(s_t^2,a_t^2) + \alpha.\delta_t^2 \quad \text{(S7}$$

This scheme can be generalized to any arbitrary number of levels of abstraction. The general equations in this case will be:

$$\delta_t^n = r_t^n + V^n(s_{t+1}^n) - Q^n(s_t^n, a_t^n) \qquad \text{(S8}$$

$$Q^n(s_t^n, a_t^n) \leftarrow Q^n(s_t^n, a_t^n) + \alpha.\delta_t^n \qquad \text{(S9}$$

Figure S2A shows schematically the neural circuit required for performing such computations for five levels of abstraction.

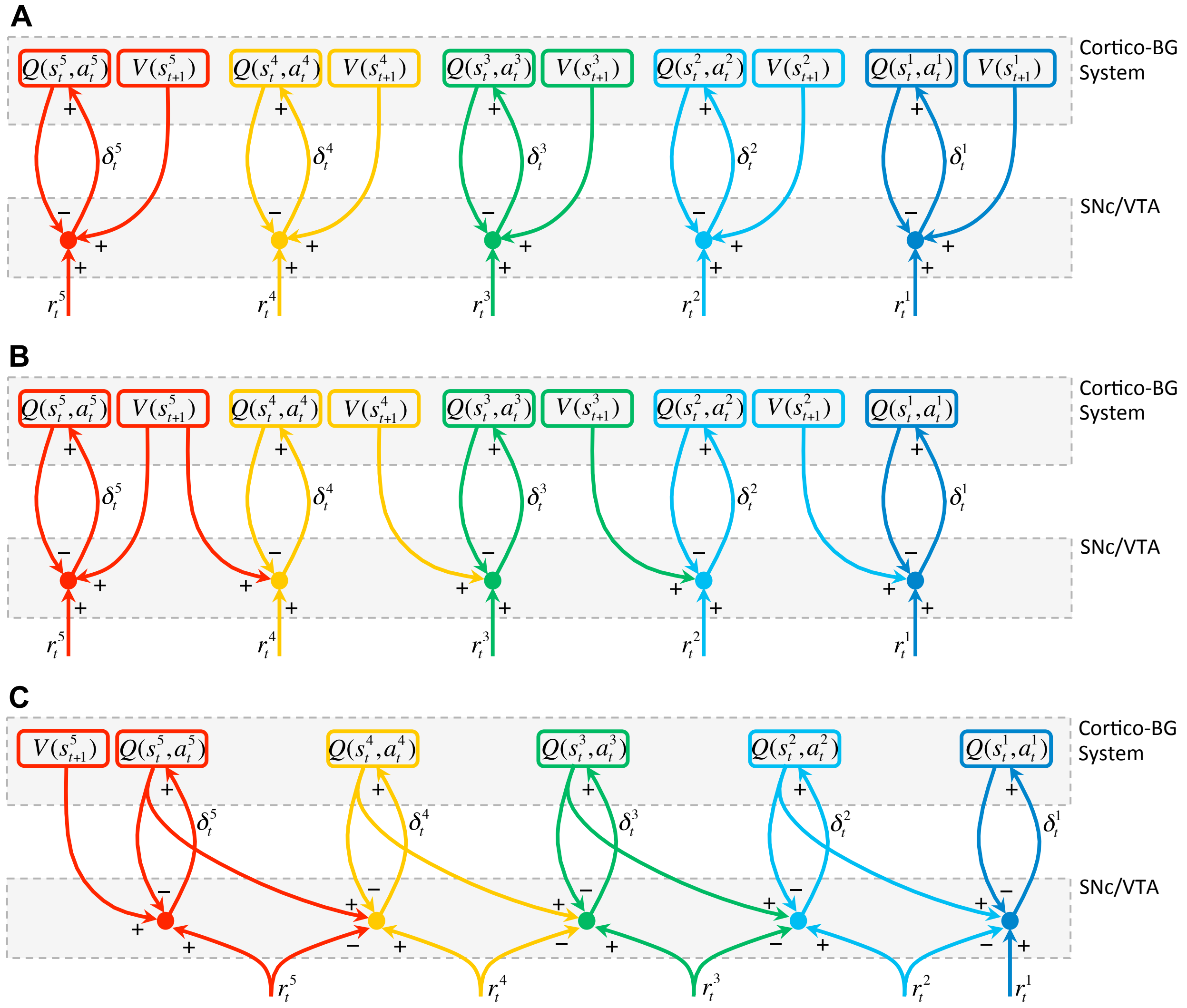

Figure S2: The corresponding neural circuit for the three discussed value learning algorithms is a hierarchical decision structure. **A,** Using a simple TD-learning algorithm (equation S7), the

prediction error signal in each level of abstraction is computed independently from other levels. **B,** In the model proposed by Haruno and Kawato (4) (equation S8), the value of the temporally-advanced state comes from one higher level of abstraction. **C,** in our model (equation S9) the value of the temporally-advanced state is substituted with a combination of the reward and Q-value of the performed action at a higher level of abstraction.

Since lower levels of the hierarchy are relatively more high-dimensional than the abstract levels, learning the optimal reward-maximizing policy becomes an increasingly hard problem as the level of abstraction decreases. However, the comparative advantage of abstract levels in rapid value-learning can be exploited by more detailed levels, through a certain way of communication between the levels.

As in Figure S1, suppose that at time t, the agent is performing the very abstraction action a_t^5, initiated from the state s_t^5. Assume that at this point, for the performance of this action to be finished, only a_t^4, which is the last piece of its corresponding sequence of actions in one lower level of abstraction, should be performed. Also, for the performance of the action a_t^4 to be finished, only a_t^3, which is the last piece of its corresponding sequence of actions in one lower level of abstraction, should be performed, and so on. Thus, after performing the primitive action a_t^1 at the lowest level, the agent enters the state s_{t+1} at all the levels. At this point, for computing the prediction error signal δ_t^1, rather than using $V^1(s_{t+1}^1)$ as in equation S2, the agent can use $V^2(s_{t+1}^2)$, since the two values converge to the same steady level. Similarly, for computing δ_t^2, $V^3(s_{t+1}^3)$ can be replaced with $V^2(s_{t+1}^2)$, and so on. Thus, in general we will have:

$$\delta_t^n = r_t^n + V^{n+1}(s_{t+1}^{n+1}) - Q^n(s_t^n, a_t^n) \qquad \text{(S10}$$

$$Q^n(s_t^n, a_t^n) \leftarrow Q^n(s_t^n, a_t^n) + \alpha.\delta_t^n \quad \text{(S11}$$

This idea first proposed by Haruno and Kawato (4) accelerates convergence at lower levels, since the value of $V^{n+1}(s_{t+1}^{n+1})$ converges faster than $V^n(s_{t+1}^n)$. A schematic neural circuit required for performing such computations is depicted in Figure S2B.

Note that these equations can be used only for updating the value of actions that finish performance of an abstract option. For other conditions, the prediction error signal will be computed as in a flat Q-learning algorithm, without being dependent on the abstract levels (as in equation S8).

It is clear from equation S10 that when the agent arrives into the state s_{t+1}^n, for computing the prediction error signal δ_t^n, the agent needs to have all the variables s_t^n, a_t^n, r_t^n, $Q^n(s_t^n, a_t^n)$, and $V^{n+1}(s_{t+1}^{n+1})$ in the working memory. However, $Q^{n+1}(s_t^{n+1}, a_t^{n+1}) - r_t^{n+1}$ can be used as an estimate of $V^{n+1}(s_{t+1}^{n+1})$, since they converge to the same steady level. As all the values of this estimate of $V^{n+1}(s_{t+1}^{n+1})$ are already available in the working memory (for computing δ_t^{n+1}), such substitution makes the algorithm more efficient in terms of working memory. With this substitution we will have:

$$\delta_t^n = r_t^n + Q^{n+1}(s_t^{n+1}, a_t^{n+1}) - r_t^{n+1} - Q^n(s_t^n, a_t^n) \quad \text{(S12}$$

$$Q^n(s_t^n, a_t^n) \leftarrow Q^n(s_t^n, a_t^n) + \alpha.\delta_t^n \quad \text{(S13}$$

In fact, in order for information to be a "trace" that can be used in active computations and control of behavior it needs to be loaded from long-term to the working memory. The working memory store is severely limited in its capacity for storage (5, 6) and forms a critical bottleneck for decision making. Therefore, for the above model to be compatible with efficient working memory storage, it is critical to decrease the number of variable required to do the valuation at

each particular step within the hierarchy. The neural circuit required for performing such computations is schematically shown in Figure S2C, which demonstrates the less dependency of this algorithm on working memory.

Note that in simulating the blocking phenomenon, we use the model described above, but in a Pavlovian experimental design, where the policy component is removed from the equations and the agent is just a passive predictor of stimulus value:

$$\delta_t^n = r_t^n + V^{n+1}(s_t^{n+1}) - r_t^{n+1} - V^n(s_t^n) \tag{S14}$$

$$V^n(s_t^n) \leftarrow V^n(s_t^n) + \alpha.\delta_t^n \tag{S15}$$

Modeling drug's effect on DA circuit

In order to model the pharmacological effect of drug on value-learning at the abstract level, a constant positive bias is added to the prediction error of the drug-seeking and –taking actions. This pharmacological effect of drug appears only in the very last primitive action of the "drug-seeking and -taking" option, when the drug is just consumed. Thus, whereas for other primitive actions the prediction error signal is computed by equation S8, only for the last primitive action of the drug-related option, the prediction error can be achieved by adding the bias D to equation S12:

$$\delta_t^n = r_t^n + Q^{n+1}(s_t^{n+1}, a_t^{n+1}) - r_t^{n+1} - Q^n(s_t^n, a_t^n) + D \tag{S16}$$

To see intuitively how the model behaves in the case of drugs, we can assume that $r_t^n = r_t^{n+1}$. This happens when the rewarding outcome of an option is received only in the last primitive action of that option, as it is the case in our simulated environment (Figure 1A). Under such assumption, equation S16 will be reduced to:

$$\delta_t^n = Q^{n+1}(s_t^{n+1}, a_t^{n+1}) - Q^n(s_t^n, a_t^n) + D \tag{S17}$$

It is clear from the above equation that $Q^n(s_t^n, a_t^n)$ will converge to $Q^{n+1}(s_t^{n+1}, a_t^{n+1}) + D$.

$$\delta_t^n = 0 \quad \Rightarrow \quad Q^n(s_t^n, a_t^n) = Q^{n+1}(s_t^{n+1}, a_t^{n+1}) + D \qquad \text{(S18}$$

Thus, the pharmacological effect of drug on the prediction error signal results in the asymptotic value of drug-seeking behavior at a level to be D units higher than that of one higher level of abstraction. Accumulation of this bias over N levels of abstraction results in the discrepancy in the asymptotic value of drug-seeking between the two extremes of the hierarchy to become $N * D$.

Figure S3 illustrates the potential sites of action of drugs that our model predicts. Note that in this figure, compared to Figure S2, the descending excitatory projection from the striatum to the more dorsal area of the midbrain is replaced with two consecutive inhibitory projections. This captures the recently demonstrated role of GABAergic interneurons of the VTA in carrying the expected reward and inhibiting the DA neurons (7).

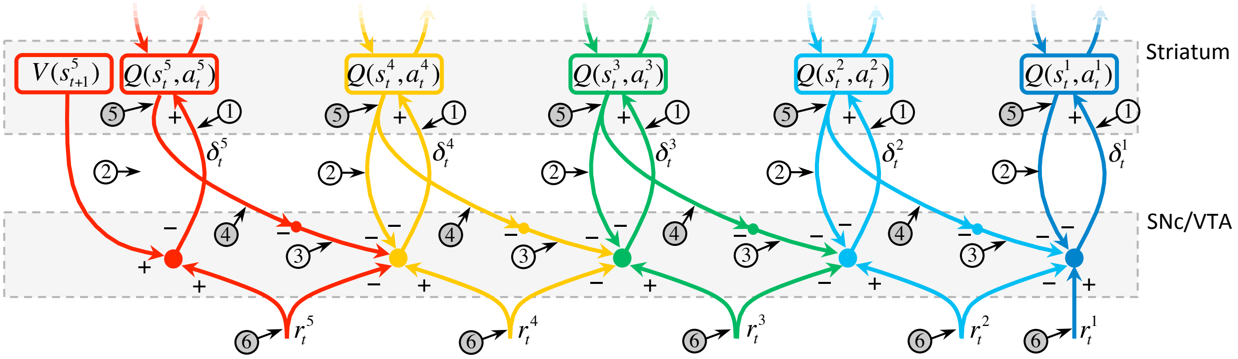

Figure S3: Our model predicts different sites of action of drugs on the reward-learning circuit: sites 1 to 3. Drugs affecting sites 4 to 6, in contrast, will not result in the behavioral and neurobiological patterns produced by simulation of the model for drugs, but will produce results similar to the case of natural rewards.

In Figure S3, an excitatory effect on site 1 results in a direct increase of the prediction error signal, δ_t^n, and thus, will produce the bias-effect that accumulates over the DA spirals and results in high values of drug-seeking response at motor levels. An excitatory effect on site 1 is equivalent to adding a positive term D to equation S12, which leads to equation S16. This is a likely mechanism of action for several drugs of abuse: Cocaine, amphetamine and ecstasy block DA reuptake and therefore, result in an increase in extracellular DA concentration (8).

Furthermore, an inhibitory effect on sites 2 and 3 will result in disinhibition of dopamine neurons and thus, will again result in cognitive/behavioral conflict. An inhibitory effect on site 2 is equivalent to subtracting a positive term D from the term $Q^n(s_t^n, a_t^n)$ in equation S12. This will result in equation S16. Similarly, an inhibitory effect on site 3 is equivalent to adding a positive term D to the term $Q^{n+1}(s_t^{n+1}, a_t^{n+1})$ in equation S12, which will again result in equation S16. This disinhibitory effect on DA neurons through GABAergic neurons appears to be the mechanism of action of several drugs (8): morphine, nicotine, alcohol, etc.

Note that an inhibitory effect on site 4 is equivalent to subtracting a positive term D from the term $Q^{n+1}(s_t^{n+1}, a_t^{n+1})$ in equation S12, which will result in the following equation:

$$\delta_t^n = r_t^n + Q^{n+1}(s_t^{n+1}, a_t^{n+1}) - r_t^{n+1} - Q^n(s_t^n, a_t^n) - D \qquad \text{(S19}$$

Clearly, this equation will not produce the simulation results showed in the paper. Furthermore, and inhibitory effect on site 5 (concurrent inhibition of sites 2 and 4) is equivalent to subtracting a positive term D from both $Q^n(s_t^n, a_t^n)$ and $Q^{n+1}(s_t^{n+1}, a_t^{n+1})$, which result in the following equation:

$$\delta_t^n = r_t^n + Q^{n+1}(s_t^{n+1}, a_t^{n+1}) - D - r_t^{n+1} - \left[Q^n(s_t^n, a_t^n) - D\right] \qquad \text{(S20}$$

$$= r_t^n + Q^{n+1}(s_t^{n+1}, a_t^{n+1}) - r_t^{n+1} - Q^n(s_t^n, a_t^n)$$

Since the two D terms cancel out one another, such effect predicts the same results as for natural rewards. This predicts that inhibiting striatal GABA neurons that project to the VTA will not produce addiction-like behavior.

Finally, an excitatory (inhibitory) effect on site 6 is equivalent to adding (subtracting) a positive term D to (from) both r_t^n and r_t^{n+1}. Thus, the two D terms will cancel out one another again and therefore, no bias will be produced in information transmission from one level of abstraction to the next.

On the relationship to the previously proposed drug-effect RL models

In the seminal paper of modeling addiction in the reinforcement learning framework (9), it was explicitly presumed that addictive drugs have a "non-compensable" pharmacological effect on the dopaminergic circuit; i.e., they never let the prediction error signal converge to zero. To capture this effect, the following functional form was suggested for modeling the effect of drug on the prediction error signal:

$$\delta_t = \max\left[r_t + V(s_{t+1}) - Q(s_t, a_t) + D,\ D\right] \qquad \text{(S21}$$

Note that since that model is based on a flat (single-layer) reinforcement learning, (s_t, a_t) in the above equation is the state-action pair that results in reward, and s_{t+1} is the temporally-advanced state. Three major criticisms that have been leveled at this assumption are that: (1:) Since the teaching signal, δ_t, never becomes zero, the value of drug-seeking choices will grow unboundedly, which is not biologically plausible. (2:) It predicts that the blocking effect should not be observed in the case of drug rewards; a prediction that has been proven untrue at least for cocaine (10). Blocking is a conditioning phenomenon where the animal is blocked from learning

an association between a stimulus and its upcoming outcome, if the outcome can be already predicted by another stimulus. In terms of reinforcement learning theory, if a stimulus can fully predict the value of its associated outcome, the prediction error signal will become zero and thus, no new learning can occur. However, according to equation S21, the prediction error signal never goes to zero for drug outcomes and thus, the animal should always be able to associate new stimuli with drug outcomes. (3:) Because of the $\max$ operator in equation S21, even if r has a large negative value, the prediction error signal will be still positive (equal to D). In fact, even if drug is paired with catastrophic consequences, the animal will still reinforce drug-seeking behavior. In other words, equation S21 predicts that animals are totally insensitive to drug-related punishments, however strong the punishments are, even at the very first time they experience drug. This prediction has been shown to be incorrect in experiments showing insensitivity of drug self-administration response to drug-associated punishments, after prolonged but not limited drug use (11).

Recently, Dezfouli et al (12) showed that the first two shortcomings mentioned above are resolved by simply assuming that the effective influence of drugs on the dopamine circuit decays after prolonged drug consumption. This assumption is based on the neurobiological data showing long-lasting reduction of dopamine D2 receptor availability in the striatum of addicts (13, 14), which results in decreased output by dopamine neurons to the downstream circuits. This assumption preserves the essential features of the original model (9) like compulsive behavior, and low elasticity of drug-seeking behavior to adverse consequences or costs.

The model we considered in this work also resolves the first two criticisms. The functional form we have adopted in this paper for modeling the effect of drug (equation S16) naturally results in the effective pharmacological influence of drugs on DA circuit to gradually converge to zero.

However, to keep the model simple, we have forgone all the mathematical complications of the model considered in (12) and instead, here the pharmacologically-induced reinforcing power of drugs simply decreases to zero within the same time-scale that the normal prediction error signal converges to zero. However, assuming a longer timescale for this process does not affect the essence of what our model predicts. We also see in the main text that the model handles correctly blocking with extensive pre-training for both drug and natural rewards.

Moreover, as we do not use a $\max$ operator, the prediction error signal does not ignore negative rewards (resolving the third shortcoming mentioned above) for the drug case. Hence, initially (early in training or drug self-administration) the model is indeed elastic to a strong punishment. However, it becomes inelastic to punishments after prolonged drug use; the reason behind that is that accumulation of drug's effect over dopamine spirals increases the drug-seeking value at motor-levels to such a high level that even a strong natural punishment will not be able to decrease it sufficiently (see main text for the central result on the valuation discordance between the abstract and detailed levels).

Uncertainty-based competition between the levels of the hierarchy: from valuation to action-policy selection

We assume that while dealing with a task with a presumably given decision hierarchy, the abstract levels control behavior during the early stages of learning the values. This is because the representation of state space is much coarser in abstract levels than in detailed levels, and learning the values becomes much faster and more flexible when the dimensionality of the space and the number of basis functions decrease (4). However, the coarse representation of actions at abstract levels makes the ultimate approximation capability inferior in those levels and thus, with more training, the control over behavior gradually shifts to more detailed levels of the hierarchy.

To model this competition mechanism among the levels of the hierarchy over controlling behavior, we can assume that at each point of time, the level that has the least uncertainty in estimating the value of available choices controls behavior. A similar mechanism was previously proposed for arbitration between the habitual and the goal-directed systems (15). In order to keep track of uncertainties, one can use Kalman Temporal Difference (KTD) (16), which in addition to learning state-action values, provides a measure of accuracy of learned values that corresponds to the certainty of estimations. In order to capture the inferior value-approximation capability of coarse representations, we introduce noise into higher levels of abstraction (known as process noise, in the KTD framework). In fact, the magnitude of the noise introduced to different levels is proportional to the abstractness of the action representation at that level.

As proof of concept, we simulated this arbitration mechanism in a simple, single-choice task, with only three levels of abstraction (Figure S4). Simulation results (Figure S5) show that during the early trials, the most abstract level has the minimum uncertainty among the levels. However, the second and then the third levels gradually achieve the minimum uncertainty-level.

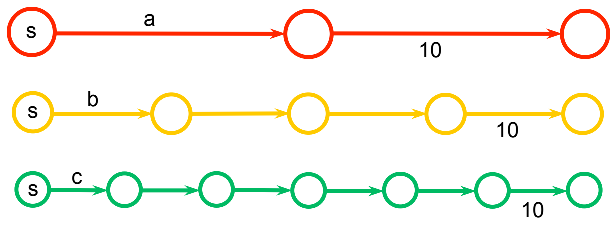

Figure S4: The task used for simulating the uncertainty-based competition mechanism among the levels of the hierarchy for taking control over behavior.

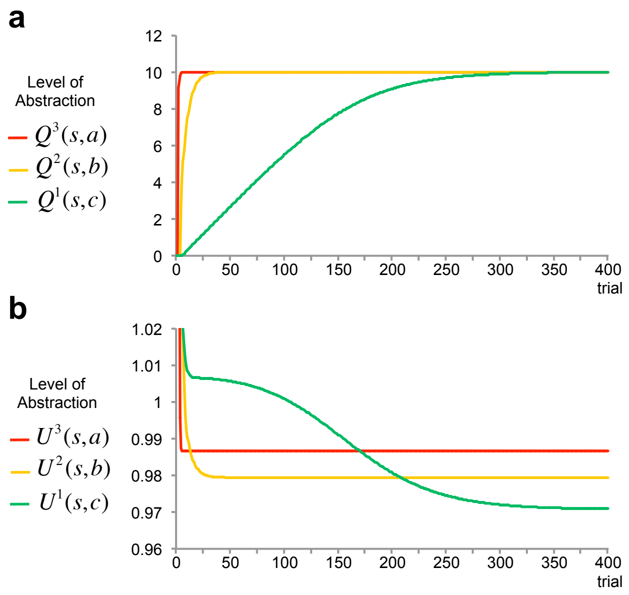

Figure S5: Simulation result, showing gradual shift of control over behavior from higher to lower levels of the hierarchy. $Q(s,a)$ and $U(s,a)$ show the estimated value and uncertainty of the state-action pairs, respectively.

Supplemental References:

1. Sutton RS, Barto AG (1998) *Reinforcement Learning: An Introduction* (MIT Press, Cambridge).

2. Doya K (2002) Metalearning and neuromodulation. *Neural networks : the official journal of the International Neural Network Society* 15:495–506.

3. Botvinick MM, Niv Y, Barto AC (2009) Hierarchically organized behavior and its neural foundations: a reinforcement learning perspective. *Cognition* 113:262–280.

4. Haruno M, Kawato M (2006) Heterarchical reinforcement-learning model for integration of multiple cortico-striatal loops: fMRI examination in stimulus-action-reward association learning. *Neural Networks* 19:1242–1254.

5. Luck SJ, Vogel EK (1997) The capacity of visual working memory for features and conjunctions. *Nature* 390:279–81.

6. Miller GA (1956) The magical number seven plus or minus two: some limits on our capacity for processing information. *Psychological review* 63:81–97.

7. Cohen JY, Haesler S, Vong L, Lowell BB, Uchida N (2012) Neuron-type-specific signals for reward and punishment in the ventral tegmental area. *Nature* 482:85–8.

8. Lüscher C, Ungless MA (2006) The mechanistic classification of addictive drugs. *{PLoS} Medicine* 3:e437.

9. Redish AD (2004) Addiction as a computational process gone awry. *Science* 306:1944–1947.

10. Panlilio LV, Thorndike EB, Schindler CW (2007) Blocking of conditioning to a cocaine-paired stimulus: testing the hypothesis that cocaine perpetually produces a signal of larger-than-expected reward. *Pharmacology, Biochemistry, and Behavior* 86:774–777.

11. Vanderschuren LJMJ, Everitt BJ (2004) Drug seeking becomes compulsive after prolonged cocaine self-administration. *Science* 305:1017–1019.

12. Dezfouli A et al. (2009) A neurocomputational model for cocaine addiction. *Neural Computation* 21:2869–2893.

13. Nader MA et al. (2006) PET imaging of dopamine D2 receptors during chronic cocaine self-administration in monkeys. *Nature Neuroscience* 9:1050–1056.

14. Volkow ND, Fowler JS, Wang G-J, Swanson JM (2004) Dopamine in drug abuse and addiction: results from imaging studies and treatment implications. *Molecular Psychiatry* 9:557–569.

15. Daw ND, Niv Y, Dayan P (2005) Uncertainty-based competition between prefrontal and dorsolateral striatal systems for behavioral control. *Nature Neuroscience* 8:1704–1711.

16. Geist M, Pietquin O, Fricout G (2009) in *Proceedings of the 2009 IEEE International Symposium on Adaptive Dynamic Programming and Reinforcement Learning (ADPRL-09)* (Nashville, USA), pp 185–192.
